# Mean amplitude of intraocular pressure excursions: a new assessment parameter for 24-h pressure fluctuations in glaucoma patients

**DOI:** 10.1038/s41433-020-0845-9

**Published:** 2020-09-24

**Authors:** Ruyi Zhai, Jingyi Cheng, Huan Xu, Zhaobin Fang, Xu Chen, Siyu Qiu, Xinghuai Sun, Richard K. Lee, Ming Xiao, Xiangmei Kong

**Affiliations:** 1 Department of Ophthalmology and Visual Science, Eye, Ear, Nose and Throat Hospital, Shanghai Medical College, Fudan University, Shanghai, China; 2 Shanghai Key Laboratory of Visual Impairment and Restoration, Fudan University, Shanghai, China; 3 NHC Key Laboratory of Myopia, Fudan University, Shanghai, China; 4 Laboratory of Myopia, Chinese Academy of Medical Sciences, Shanghai, China; 5 Department of Ophthalmology, Shanghai Jing’an District Beizhan Hospital, Shanghai, China; 6 State Key Laboratory of Medical Neurobiology, Institutes of Brain Science and Collaborative Innovation Center for Brain Science, Fudan University, Shanghai, China; 7 Bascom Palmer Eye Institute, University of Miami Miller School of Medicine, Miami, FL USA

**Keywords:** Medical research, Glaucoma

## Abstract

**Background:**

Intraocular pressure (IOP) is important in the pathogenesis of glaucoma and its circadian fluctuations are important in the disease management; however, there are no adequate parameters to describe the fluctuations. This study investigates a new parameter, mean amplitude of intraocular pressure excursion (MAPE), and compares its ability in assessing 24-h IOP fluctuations with other ocular parameters.

**Methods:**

Only the right eye was evaluated in each of the 79 healthy people and 164 untreated patients with primary open angle glaucoma (POAG). Each participant underwent 24-h IOP monitoring by measuring IOP every 2 h. IOP fluctuations were expressed as MAPE calculations and currently used parameters included mean IOP, standard deviation of IOP, max difference and area under the circadian IOP curve. Comprehensive ophthalmologic examinations were also performed. Associations between visual field deficits and IOP fluctuation parameters were investigated via partial least squares (PLS) regression. Diagnostic performance was evaluated with area under the receiver operating characteristic curves (ROC).

**Results:**

Compared with healthy volunteers, the MAPE values in POAG patients were higher (4.16 ± 1.90 versus 2.45 ± 0.89, *p* < 0.01). In PLS regressions where visual field deficits were as dependent variable, MAPE had the highest score regarding variable importance in projection, and its standard regression coefficient was larger than other parameters. Diagnostic performance analysis showed the area under ROC of MAPE for glaucoma detection was 0.822 (0.768–0.868, *p* < 0.001).

**Conclusions:**

MAPE might be an effective parameter in clinic to characterise IOP circadian fluctuations.

## Introduction

Elevated intraocular pressure (IOP) is the most important modifiable risk factor for the development of glaucoma [1]. IOP changes dynamically—fluctuating according to human bio-rhythms, position, sleep and other factors. The influence of IOP on the optic nerve can be considered as two aspects: (1) the duration and magnitude of chronic sustained ocular hypertension, and (2) the short-term fluctuations of IOP during a 24-h period. The former is mostly assessed though single IOP measurement during each office visit and is the most widely used measurement of IOP in the long-term management of glaucoma treatment. However, this measurement does not profile an individual’s IOP fluctuation pattern comprehensively or physiologically. There were previous researches demonstrating that long-term IOP fluctuation is significantly related to the risk of glaucoma progression [2, 3]. Twenty-four-hour IOP monitoring [4–8] and its association with glaucomatous optic neuropathy needs further investigation.

Twenty-four-hour IOP fluctuations are also called ‘circadian fluctuations’ or ‘short-term fluctuations’, while IOP fluctuations that occur over months or years are called ‘long-term fluctuations’ [9]. Importantly, it is unknown which type of IOP parameter, IOP (average or peak value) or IOP fluctuations, contributes more to glaucoma progression [2, 3, 10–14]. Differences in measuring and understanding these IOP values can be attributed to research design, statistical analysis, study population and IOP measurement methodology.

Also, the current clinically relevant definition of IOP fluctuation is confusing, non-uniform, and may not accurately reflect its role in glaucoma [9]. To study circadian IOP fluctuations, some researchers have investigated the use of contact lens sensors (CLSs), which measure IOP-related patterns continuously over 24 h [15, 16]. However, CLSs are not available for most patients when compared with traditional 24-h IOP monitoring.

Clinical measurements assessed from 24-h IOP monitoring include different time-point IOP values and parameters such as mean, peak, trough and maximum difference of one’s 24-h IOP value. These parameters provide a general description of the individual’s IOP variation. Lack of accurate and more complete characterisation of circadian IOP fluctuation relative to visual field (VF) progression not only makes assessment difficult, but also limits insight into the role of 24-h IOP on optic nerve function. Typically, only the peak value and the maximum IOP difference are used in clinically.

Circadian fluctuations occur not only with IOP, but also in blood glucose. The gold standard to understand 24-h fluctuations in blood glucose, mean amplitude of blood glucose excursion (MAGE) [17], has been widely used clinically. Considering the similarity between fluctuations of IOP and blood glucose, and how variable blood glucose with acute fluctuations also contributes independently to diabetes mellitus severity [18], it is reasonable to introduce the principle of MAGE into 24-h monitoring of IOP and the severity of glaucoma.

To better describe the instability of circadian IOP, the present study describes a new parameter, mean amplitude of IOP excursion (MAPE), based on the principles of MAGE and calculated by different time-point IOP values throughout 24-h IOP monitoring. Aiming to investigate whether MAPE could better reflect circadian IOP fluctuation than present parameters, the study also brought relationships between IOP fluctuations and severity of glaucoma into study.

## Materials and methods

This research protocol was reviewed and approved by the medical ethics committee of the Eye and Ear, Nose and Throat (ENT) Hospital of Fudan University. Written informed consent was obtained from all participants. The study was conducted following the tenets of the Declaration of Helsinki.

### Study populations

This cross-sectional study recruited healthy volunteers and untreated POAG patients at the Eye and ENT Hospital of Fudan University. All subjects underwent a comprehensive eye examination, including best corrected visual acuity, IOP measurement (Goldmann applanation tonometry), slit-lamp biomicroscopy, gonioscopy, funduscopy, central corneal thickness (CCT) and axial length (AL) (IOL Master, Carl Zeiss Inc. Jena, Germany). Glaucoma patients also undergo VF testing (Humphrey automated perimetry or Octopus automated perimetry). Only the right eyes of the volunteers and patients were selected for the study.

### Inclusion and exclusion criteria for the patient group

Individuals more than 20 years old with a confirmed POAG diagnosis were included in the patient group. A POAG diagnosis included assessment of open and wide anterior chamber angles in both eyes, characteristic VF deficits by Humphrey automated perimetry (a cluster of at least three non-edge points with *P* < 0.05, and *P* < 0.01 for one point in the pattern deviation plot, mean defect and PSD exceeding the 95% confidence interval, and glaucoma hemifield test result beyond the normal range) or by OCTOPUS 101 automated perimetry (one point decreased by 10 dB, or two adjacent points decreased by 5 dB, or three adjacent points decreased by 2 dB), and typical glaucomatous optic discs with at least two of the following characteristics: cup-to-disc (C/D) ratio ≥0.6, C/D asymmetry >0.2, disc haemorrhage, localised rim notch or loss of retinal nerve fibre layer related to glaucoma.

Patients with a history of anti-glaucoma surgery including laser treatment, anti-glaucoma medication use within a month of the study, unreliable VF examinations with fixation errors > 20%, false positives > 15% or false negatives > 33% were excluded. Patients with other ocular or systemic diseases known to cause VF deficits or optic nerve damage were also excluded. Also, patients with ocular inflammation or trauma, or other secondary causes, leading to ocular hypertension in one or both eyes and patients with IOP measurements over 30 mmHg were excluded.

### Inclusion and exclusion criteria for healthy volunteers

Individuals without glaucoma and with open and wide anterior chamber angles, a normal cup-to-disc ratio, optic nerve head form, and retinal nerve fibre layer thinness in both eyes were considered healthy volunteers. However, individuals with a history of other ophthalmic diseases known to cause IOP elevation or family history of glaucoma in first-degree relatives were excluded.

### 24-h IOP monitoring

All subjects underwent 24-h IOP monitoring at Beizhan Hospital using published methods [4, 8]. In brief, participants were asked to maintain normal biological activities and rhythms, and were housed in special area of the hospital a day in advance of the 24-h recording. IOP measurements were taken from 8:00 AM to 6:00 AM the next day every 2 h with a non-contact tonometer (NIDEK, Japan) by the same experienced operator. Lights were turned off to promote sleep from 10:00 PM to 6:00 AM. During this time, participants were awakened at 2-h intervals and measured immediately in the upright position. At each time point, IOP measurements were taken three times and the average value was used for analysis without CCT correction.

### Measurement of fluctuation parameters

#### Mean amplitude of IOP excursions calculation

**Fig. 1:**
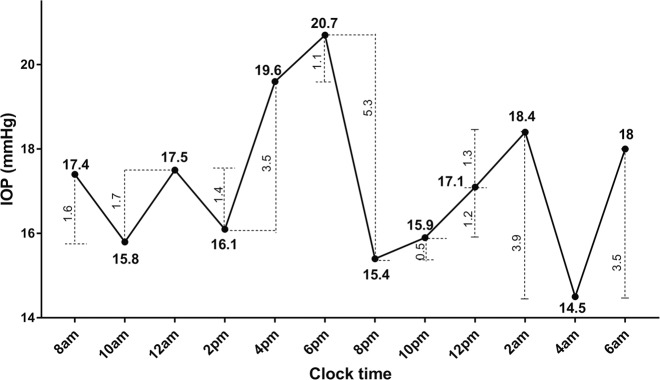
Circadian intraocular fluctuation of one primary open angle glaucoma patient. The standard deviation of the patient’s circadian IOP was 1.8 mmHg. IOP intraocular pressure.

MAPE determines the IOP fluctuations exceeding a certain limit of each subject, or ‘effective fluctuations’, by identifying large IOP fluctuations and ignoring trivial ones. Like the principle of ‘MAGE’, the limit of MAPE was also set as 1 SD value of each subject’s circadian IOP value. MAPE was calculated as the arithmetic mean value of the relevant IOP fluctuations meeting this criterion. There is one example for calculating MAPE. Figure 1 shows circadian IOP fluctuation of a typical POAG patient whose SD value is 1.8 mmHg for the day. By calculating the difference between two adjacent values, we can derive a series of excursion values. As shown in Fig. 1, the first excursion, from 17.4 to 15.8 mmHg, is 1.6 mmHg, less than one SD value 1.8 mmHg. When an IOP limit was set as one SD, according to the definition, the first excursion was not an effective IOP fluctuation. Thus, only the fourth excursion (3.5 mmHg, from 16.1 to 19.6 mmHg), the sixth excursion (5.3 mmHg, from 20.7 to 15.4 mmHg), the tenth excursion (3.9 mmHg, from 18.4 to 14.5 mmHg) and the eleventh excursions (3.5 mmHg, from 18.4 to 18 mmHg) were considered relevant IOP fluctuations among 24-h IOP measurements of the patient. Based upon this analysis, MAPE by the limit line for one SD is 4.05 mmHg.

Another new parameter, area under circadian IOP curve (AUC_IOP), was calculated by averaging the area between IOP fluctuation curves by connecting IOP values at each time point and basal IOP (0 mmHg) every 2 h. Other previously existing parameters were as follows: SD of IOP, obtained from all time-points during the 24-h recording; max difference of IOP, calculated by subtracting the trough IOP from the peak IOP; and peak IOP and trough IOP, noted as the highest and lowest values among the twelve IOP values of circadian IOP curve drawn, respectively.

### Statistical analyses

The differences between untreated primary open angle glaucoma (POAG) patients and healthy volunteers were compared. The respective associations of MAPE values and previous fluctuation parameters for glaucoma severity shown by pattern standard deviation (PSD), a marker of glaucomatous VF deficits [19] were investigated by Partial least squares (PLS). Also, the study evaluated the diagnostic efficiency of MAPE and other IOP fluctuation parameters for POAG versus normality.

Independent *t*-tests and Mann-Whitney statistical tests were used to compare normally distributed and non-normally distributed data, respectively. Chi-square analysis was used to compare categorical data. Pearson correlation was used to test collinearity among fluctuation parameters. The statistical analyses were performed using SPSS 20 (IBM Corp., New York, NY). PLS regression was used for multivariate analysis, which provides regression coefficients and variable importance in projection (VIP) scores. VF deficit parameter, PSD were used as the dependent variable. The diagnostic performance of fluctuation parameters was measured and compared by receiver operating characteristic (ROC) using statistical software MedCalc (version 11.4). Statistical significance was accepted at *p* < 0.05 for 2-tailed tests.

## Results

### Demographics and ophthalmic characteristics of healthy people and POAG patients

A total of 243 individuals were included in this study: 79 healthy controls and 164 POAG patients were enrolled to study circadian fluctuations. The average age of the subjects in healthy controls and POAG groups was 48.3 ± 15.18 and 49.0 ± 14.01 (*p* = 0.755), respectively. Males accounted for 34.18% in healthy group and 42.07% in POAG group (*p* = 0.238). Compared with healthy participants, POAG patients had higher mean IOPs (13.43 ± 2.57 versus 17.36 ± 4.43), greater vertical cup-disk ratios (0.52 ± 0.12 versus 0.77 ± 0.45) and worse visual acuity (1.10 ± 0.24 versus 0.76 ± 0.30) with statistical significance (*p* < 0.001). No difference was measured in CCT between the two groups (Table 1).

**Table 1:** Clinical characteristics of healthy individuals and POAG patients.

	Healthy Individuals	POAG Patients	*p*-value
Number	79	164	
Age (mean, SD)	48.30 (15.18)	49.00 (14.01)	0.755
Gender (male, %)	27 (34.18)	69 (42.07)	0.238
C/D ratio	0.52 (0.12)	0.77 (0.45)	<0.001
VA	1.10 (0.24)	0.76 (0.30)	<0.001
CCT (um)	540.59 (25.81)	540.42 (34.44)	0.966
AL (mm)	24.67 (1.70)	25.32 (2.17)	0.012
Mean IOP (mmHg)	13.43 (2.57)	17.36 (4.43)	<0.001
Peak IOP (mmHg)	15.96 (2.69)	21.61 (5.45)	<0.001
Trough IOP (mmHg)	11.03 (2.53)	13.95 (3.82)	<0.001
SD of IOP (mmHg)	1.49 (0.47)	2.37 (0.97)	<0.001
Max difference (mmHg)	4.93 (1.63)	7.60 (3.00)	<0.001
AUC_IOP	294.40 (56.88)	379.40 (96.42)	<0.001
MAPE (mmHg)	2.45 (0.89)	4.16 (1.90)	<0.001

Data are expressed as mean (SD) unless otherwise specified.

*POAG* primary open angle glaucoma, *C/D ratio* cup-to-disk ratio, *VA* visual acuity, *CCT* central corneal thickness, *AL* axial length, *MAPE* mean amplitude of IOP excursions, *SD* standard deviation, *AUC_IOP* area under curve of circadian IOP.

### Comparison of circadian IOP fluctuations

The newly proposed parameters including MAPE and AUC_IOP as well as other IOP parameter measurements such as the mean, peak value, trough value, max difference and SD are displayed in Table 1. According to the definition of MAPE, the boundary line was calculated. Among POAG patients, a marked high level was seen in MAPE (4.16 ± 1.90 versus 2.45 ± 0.89) and AUC_IOP (379.40 ± 96.42 versus 294.40 ± 56.88) when compared with healthy volunteers (*p* < 0.001). Similarly, POAG patients have higher max difference and SD of IOP (*p* < 0.001).

### Associations between VF deficits and mean amplitude of IOP excursions

**Fig. 2:**
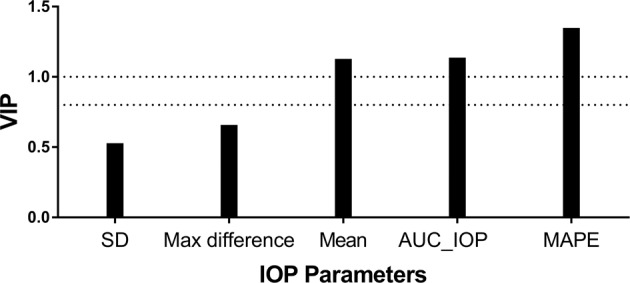
Variable importance in projection scores for the predictors in PLS partial least squares regression. VIP variable importance in projection, PLS partial least squares, PSD pattern standard deviation, SD standard deviation, AUC_IOP area under curve of circadian IOP, MAPE mean amplitude of IOP excursions.

Collinearity tests showed a significant correlation among all fluctuation parameters (*p* < 0.05; Supplementary Table S1). Among POAG patients only patients undergoing Humphrey VF tests with PSD values were enrolled in the next PLS regression (*n* = 47). PLS regression for multivariate analysis including MAPE, AUC_IOP, mean value of IOP, SD of IOP and IOP max difference were performed to study the independent correlations of IOP fluctuation parameters and VF deficits (PSD). MAPE was the most important fluctuation parameter based on VIP values, while parameters such as the SD of IOP and max difference did not reach the criterion (0.8) (Fig. 2); VIP scores below 0.8 signified low importance. PLS regression results showing associations between fluctuation parameters and PSD were shown in Table 2. The standard regression coefficient of MAPE was 0.533, which was the largest, while the standard regression coefficients of other parameters did not exceed 0.2.

**Table 2:** Independent effects of MAPE on visual deficits by partial least squares regression.

	Coefficient	Standard regression coefficient
Mean IOP	−0.226	−0.136
SD of IOP	−0.141	−0.023
Max difference	−0.308	−0.160
AUC_IOP	−0.009	−0.116
MAPE	1.750	0.533

The dependent variable is the pattern standard deviation data for the enrolled patients.

*MAPE* mean amplitude of IOP excursions, *IOP* intraocular pressure, *SD* standard deviation, *AUC_IOP* area under curve of circadian IOP.

### Diagnosis efficiency

**Fig. 3:**
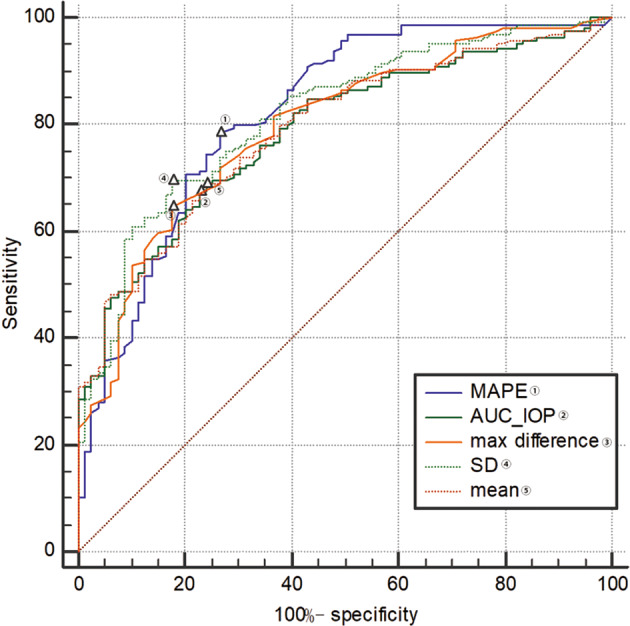
Diagnosis efficiency of mean amplitude of intraocular pressure excursions. Number of healthy volunteers and POAG patients were 79 and 164, respectively. MAPE mean amplitude of IOP excursions, SD standard deviation, AUC_IOP area under curve of circadian IOP, POAG primary open angle glaucoma. △, a label of the position correlating to the Youden’s index.

ROC curves were performed to compare the two groups including 79 healthy volunteers and 164 POAG patients. The ROC curves of MAPE, AUC_IOP, max difference, SD and mean of IOP generated an area under the curve of 0.822 (95% CI, 0.768–0.868), 0.788 (0.731–0.838), 0.797 (0.740–0.845), 0.817 (0.763–0.864) and 0.792 (0.735–0.841), respectively (all *p* < 0.01; shown in Fig. 3 and Supplementary Table S2). However, no significant difference was seen between diagnosis efficiency of MAPE and other parameters (*p* > 0.05).

## Discussion

This study proposes a new parameter, MAPE, which quantifies short-term fluctuations during a 24-h period in a manner clinically similar to detecting blood glucose fluctuations to assess glucose control in diabetics. In untreated POAG patients, the MAPE amplitude was larger when compared with healthy people. Moreover, in PLS regression analysis including MAPE, AUC_IOP, SD, max difference and mean value, MAPE showed the largest VIP scores and the largest standard regression coefficients when associated with visual function (measured by PSD). Thus, MAPE describes circadian IOP fluctuations better than the current IOP fluctuation parameters.

The principle of MAPE was derived from the current golden standard, mean amplitude glucose excursions (MAGE), to assess acute blood glucose fluctuations [18, 20]. Many similarities exist between fluctuation patterns of IOP and blood glucose relative to glaucoma and diabetes, respectively. IOP and glucose fluctuate during the day and are associated with disease status for glaucoma and diabetes mellitus, respectively. Similar to the principles of MAGE [17], the principles of MAPE are ‘filtering’ IOP fluctuations and highlighting the important IOP fluctuations while de-emphasising the subtle IOP excursions, better reflecting the circadian IOP fluctuations. However, due to characteristics of IOP, some details were unlike MAGE for blood glucose. First, for the definition of fluctuation, we used the changing IOP value during each adjacent time period (2 h), namely the rate of IOP change (mmHg per 2 h), instead of the difference between the adjacent peak and valley values. MAGE is assessed by continuous glucose monitoring systems, which could produce a series of continuous blood glucose values but our 24-h IOP monitoring measured IOP every 2 h, only offering 12 time points. Using the difference between peak and valley values would reduce available information and introduce more bias. For instance, as shown in Fig. 1, if we use the MAGE algorithm, we would only get 4 (from nadir to peak or from peak to nadir) fluctuations. Second, MAGE calculations only use the direction of the first valid fluctuation (rise or fall) [17]. This is, if the first valid fluctuation is from peak to nadir, then MAGE only identifies fluctuations from peak to nadir, excluding valid fluctuations from nadir to peak. As only one direction is counted, half of the data is lost. Also, MAGE has difficulty in determining the start and end points of the curve change needed to calculate differences from nadir to peak or from peak to nadir; different starting points would alter the results. Therefore, when we calculated MAPE, we included effective IOP fluctuations in both directions. Although much has been reported regarding 24-h IOP patterns in healthy people and glaucoma patients, to our knowledge this is the first time the MAGE model of blood glucose monitoring has been used to understand IOP fluctuations and glaucoma. We observed an association between 24-h IOP fluctuation and VF deficits using 24-h IOP monitoring.

Before performing further multivariate analysis associated with PSD, we tested correlations among fluctuation parameters and found significant correlations between any two parameters. Considering the high collinearity among fluctuation parameters that could influence prediction method performance, PLS regression is more suitable than a multivariate linear approach in multivariate analysis [21]. In addition to collinearity, PLS regression would perform better when there are many independent variables and relatively insufficient sample data [22]. In general, standard regression is appropriate when the sample size is 10–20 times the number of independent variables. Our study did not meet the criteria for standard regression analysis because there were 5 variables and only 47 participates underwent Humphrey automated perimetry. Using PLS regression, we calculated VIP scores by estimating the importance of each variable and regression coefficient. VIP scores near or greater than 1 are considered more important and scores below 0.8 are of lower importance; with high correlation, the proper cutoff VIP value may be higher than 1 [23]. In our models, MAPE showed the greatest VIP scores and standard coefficients. However, VIP scores of SD and max difference, two of the most used parameters to describe 24-h IOP fluctuations, did not reach 0.8. The current parameters including mean IOP, SD of IOP and max difference between peak and valley values showed little correlation with visual deficit, which may explain why previous studies using SD [2, 14], max difference [3, 10, 11] or mean of IOP [3] demonstrated conflicting results of correlation between IOP fluctuation and glaucomatous VF loss. Importantly, reviewing the calculation of AUC_IOP, we found that there was a high correlation in calculation formula between MAPE and AUC_IOP, verified by correlation analyses (shown in Supplementary Table S1). Therefore, we did not consider it as a new effective parameter.

In addition, using a CLS system, De Moraes et al. first found that some CLS parameters were associated with glaucoma progression (*p* < 0.05) [24, 25], which is meaningful to study circadian IOP. Parameters from CLS recordings also proved to assess treatment efficacy [26, 27]. However, CLS parameters are not measured IOP values but signal output differences captured by ocular dimensional changes [28]. Furthermore, a weak correlation between CLS parameters and IOP measurements taken by Tonopen tonometry has been suggested [29]. Also, participants in these particular studies were treated glaucoma patients, which may affect IOP profiles and VF progression. To exclude the effect of therapeutic intervention on IOP and VF examination, we only enrolled untreated patients or patients who had discontinued medications for more than 1 month and underwent a medication washout period. However, CLS as a non-invasive sensor to continually monitor IOP, has many advantages over traditional 24-h tonometry. It is possible to combine the concept of MAGE with CLS in the future.

The study has some limitations. First, it is subject to the inherent limitations of cross-sectional study designs, so it must be highlighted that the study also does not address any causal relationships between MAPE and glaucoma progression. Prospective studies on the association between MAPE and the progression of glaucomatous visual deficits are warranted. Limitations also exist with 24-h IOP monitoring. The sitting position does not mimic the physical position during sleep and rising from sleep may affect the IOP, although we examined IOP immediately when participants were awakened and sitting upright. In addition, though GAT is the gold standard to test IOP, patients in our study were monitored by non-contact tonometer. However, some study also proved that there is moderate inter-instrument consistency between the non-contact tonometer and GAT [30]. The axial length is statistically different between healthy volunteer group and POAG patients group. Many previous studies have reported that myopia is an important independent risk factor for POAG [31–34], which could explain why the axial length of the POAG group was significantly longer than that of the normal population. In addition, previous studies also suggest that long axial length and high IOP may play a joint role in the progress of glaucoma [35]. On the other hand, Wilson et al. [36] found that both axial length and IOP fluctuate during the day but there were no correlations in the amplitude, period or phase of the two rhythms. Therefore, we believe that although the axial lengths of the two groups are different, this will not affect the IOP and IOP fluctuations of the two groups, which would interfere with our results.

In summary, this study proposes a new parameter, MAPE, to characterise circadian IOP patterns in healthy people and untreated POAG patients. In untreated POAG patients, MAPE is more closely correlated with glaucoma severity than current IOP fluctuation parameters. This new parameter based on a principle of ‘filtering’ IOP fluctuations may better illustrate 24-h IOP fluctuations.

### Summary

#### What was known before

- IOP is important in the pathogenesis of glaucoma and its circadian fluctuations are important in the disease management; there are no adequate parameters to describe the fluctuations.

#### What this study adds

- The new parameter, MAPE might be an effective parameter in clinic to characterise IOP circadian fluctuations.

## Supplementary information

Supplemental Tables
