# Supplementary material for: Mean amplitude of intraocular pressure excursions: a new assessment parameter for 24-h pressure fluctuations in glaucoma patients
Source: Eye (Lond). 2020 Sep 24;35(1):326–33. doi: 10.1038/s41433-020-0845-9 (PMC7852691; doi:10.1038/s41433-020-0845-9)
Supplement: Supplementary file 1 — Supplemental Tables [file 41433_2020_845_MOESM1_ESM.docx]

Table S1. Correlation analyses testing collinearity between parameters

|  | SD | Max difference | AUC_IOP | MAPE |
| --- | --- | --- | --- | --- |
| Mean | 0.430^**^ | 0.429^**^ | 0.999^**^ | 0.396^**^ |
| SD |  | 0.968^**^ | 0.416^**^ | 0.938^**^ |
| Max difference |  |  | 0.415^**^ | 0.894^**^ |
| AUC_IOP |  |  |  | 0.384^**^ |

Abbreviations: IOP, intraocular pressure; MAPE, mean amplitude of IOP excursions; AUC_IOP, area under curve of circadian IOP. *, *p* value <0.05; **, *p* value <0.01.

## Table S2. Diagnosis efficiency of the mean amplitude of intraocular pressure excursion parameter for primary open angle glaucoma

|  | AUC | 95% CI | P value | Cutoff value | | | | |
| --- | --- | --- | --- | --- | --- | --- | --- | --- |
|  |  |  |  | Cutoff value | Se | Sp | Youden index J | |
| MAPE | 0.822 | 0.768-0.868 | <0.0001 | 2.78 | 0.787 | 0.734 | | 0.521 |
| AUC_IOP | 0.788 | 0.731-0.838 | <0.0001 | 335.7 | 0.677 | 0.772 | | 0.449 |
| Max difference | 0.797 | 0.740-0.845 | <0.0001 | 6 | 0.646 | 0.823 | | 0.469 |
| SD | 0.817 | 0.763-0.864 | <0.0001 | 2.78 | 0.787 | 0.734 | | 0.521 |
| Mean | 0.792 | 0.735-0.841 | <0.0001 | 1.80 | 0.695 | 0.823 | | 0.518 |

Abbreviations: IOP, intraocular pressure; CI, confidence interval; AUC, area under receiver operating characteristic; Se, sensitivity; Sp, specificity; MAPE, mean amplitude of IOP excursions; AUC_IOP, area under curve of circadian IOP; SD, standard deviation of one’s 24-hour intraocular pressure values. Number of healthy volunteers and POAG patients were 79 and 164, respectively.
